# Prevalence of Cryptococcal Antigenemia and Cost-Effectiveness of a Cryptococcal Antigen Screening Program – Vietnam

**DOI:** 10.1371/journal.pone.0062213

**Published:** 2013-04-23

**Authors:** Rachel M. Smith, Tuan Anh Nguyen, Hoang Thi Thanh Ha, Pham Hong Thang, Cao Thuy, Truong Xuan Lien, Hien T. Bui, Thai Hung Le, Bruce Struminger, Michelle S. McConnell, Robyn Neblett Fanfair, Benjamin J. Park, Julie R. Harris

**Affiliations:** 1 Epidemic Intelligence Service, Scientific Education and Professional Development Program Office, Centers for Disease Control and Prevention, Atlanta, Georgia, United States of America; 2 Division of Foodborne, Waterborne, and Environmental Diseases, National Center for Emerging and Zoonotic Infectious Diseases, Centers for Disease Control and Prevention, Atlanta, Georgia, United States of America; 3 National Institute for Hygiene and Epidemiology, Hanoi, Vietnam; 4 National Hospital of Tropical Diseases, Hanoi, Vietnam; 5 Pasteur Institute, Ho Chi Minh City, Vietnam; 6 Division of Global HIV/AIDS, Centers for Disease Control and Prevention, Hanoi, Vietnam; 7 Division of Global HIV/AIDS, Centers for Disease Control and Prevention, Ho Chi Minh City, Vietnam; Institute of Infectious Diseases and Molecular Medicine, South Africa

## Abstract

**Background:**

An estimated 120,000 HIV-associated cryptococcal meningitis (CM) cases occur each year in South and Southeast Asia; early treatment may improve outcomes. The World Health Organization (WHO) recently recommended screening HIV-infected adults with CD4<100 cells/mm^3^ for serum cryptococcal antigen (CrAg), a marker of early cryptococcal infection, in areas of high CrAg prevalence. We evaluated CrAg prevalence and cost-effectiveness of this screening strategy in HIV-infected adults in northern and southern Vietnam.

**Methods:**

Serum samples were collected and stored during 2009–2012 in Hanoi and Ho Chi Minh City, Vietnam, from HIV-infected, ART-naïve patients presenting to care in 12 clinics. All specimens from patients with CD4<100 cells/mm^3^ were tested using the CrAg lateral flow assay. We obtained cost estimates from laboratory staff, clinicians and hospital administrators in Vietnam, and evaluated cost-effectiveness using WHO guidelines.

**Results:**

Sera from 226 patients [104 (46%) from North Vietnam and 122 (54%) from the South] with CD4<100 cells/mm^3^ were available for CrAg testing. Median CD4 count was 40 (range 0–99) cells/mm^3^. Nine (4%; 95% CI 2–7%) specimens were CrAg-positive. CrAg prevalence was higher in South Vietnam (6%; 95% CI 3–11%) than in North Vietnam (2%; 95% CI 0–6%) (p = 0.18). Cost per life-year gained under a screening scenario was $190, $137, and $119 at CrAg prevalences of 2%, 4% and 6%, respectively.

**Conclusion:**

CrAg prevalence was higher in southern compared with northern Vietnam; however, CrAg screening would be considered cost-effective by WHO criteria in both regions. Public health officials in Vietnam should consider adding cryptococcal screening to existing national guidelines for HIV/AIDS care.

## Introduction

Cryptococcal meningitis (CM) is one of the most common opportunistic infections (OI) among HIV-infected individuals, with an estimated 1 million cases of HIV-associated CM and 600,000 deaths each year [1]. Of those, an estimated 120,000 CM cases and 66,000 deaths occur in South and Southeast Asia [1], making CM one of the three most common HIV-associated OIs [2], [3], [4] in this region. Despite access to appropriate antifungal treatment, CM mortality in this region is between 40–55% [1], [5], [6], considerably higher than CM mortality in the developed world [1], [7].

Reducing CM mortality has long been a focus of HIV care and treatment programs; however, recently the focus has shifted from improving CM treatment to preventing symptomatic CM through early cryptococcal disease detection and pre-emptive treatment. CM represents a disseminated form of cryptococcal disease that requires hospitalization, with costly drug regimens (including amphotericin B) that have substantial side effects. Although early infection is treatable with relatively inexpensive and non-toxic drugs (typically oral fluconazole), it may be asymptomatic and thus go unnoticed. Cryptococcal antigen (CrAg), a biologic marker of cryptococcal infection, is detectable in sera a median of 3 weeks (range 5–234 days) before symptoms of meningitis appear [8], and is most commonly found in patients with CD4<100 cells/mm^3^ [9]. Otherwise healthy HIV-infected persons with detectable serum CrAg have increased mortality when compared to their CrAg-negative counterparts [10], [11]; pre-emptive treatment of serum CrAg-positive patients with fluconazole and anti-retroviral therapy (ART) has been shown, in a small observational study, to improve survival [12], compared with ART alone, and has been recommended for consideration by the World Health Organization (WHO) [13]. This period of asymptomatic antigenemia before symptomatic meningitis provides a window of opportunity to treat patients and potentially prevent fatal cryptococcal disease.

Use of CrAg detection tests in resource-limited regions has been limited by the expense and laboratory infrastructure required. However, the recent development of an inexpensive, easy-to-use, highly sensitive and specific [14] dipstick CrAg detection test called the lateral flow assay (LFA) (Immy, Norman, Oklahoma, USA) may increase accessibility of CrAg testing for clinicians in resource-limited settings. In 2011, the WHO released guidelines for diagnosis, prevention and management of cryptococcal disease, which recommended consideration of serum CrAg-based screening for early cryptococcal infection using antigen-based tests, including the LFA [13]. The target population for screening is HIV-infected persons with a CD4<100 cells/mm^3^ living in areas with a high prevalence of cryptococcal disease [13]. However, the circumstances under which CrAg screening programs are cost-effective are country-specific, as they depend not only on prevalence of cryptococcal disease, but also local drug costs and other aspects of treatment. Existing data demonstrating the cost-effectiveness of CrAg screening programs are limited to studies from Uganda [12], [15], where costs and CrAg prevalence differ from those in Southeast Asia, and Cambodia [16], where a model with inputs that differ substantially from the WHO-recommended cryptococcal screening strategy was utilized.

To date, two small studies have evaluated the serum CrAg prevalence among high-risk (CD4<100 cells/mm^3^) HIV-infected patients in Southeast Asia: in Thailand, the observed prevalence was 13% [9], and in Cambodia, 21% [17]. In Vietnam, situated near both Cambodia and Thailand, between 200,000–350,000 persons were projected to be living with HIV/AIDS by 2012 [18]. Although a small number of studies have described the burden of cryptococcal meningitis in Vietnam [2], [19], [20], the prevalence of serum CrAg positivity among HIV-infected individuals is not known. We evaluated the prevalence of serum CrAg in Vietnam among HIV-infected persons with CD4<100 cells/mm^3^, and modeled the cost-effectiveness of an in-country screening program.

## Methods

### Study Enrollment and Specimen Testing

The BED-assay study (an HIV incidence validation study which used the BED-capture enzyme immunoassay to estimate time since HIV infection [21]) and HIV-DR (HIV Drug Resistance) monitoring study, both conducted in Vietnam, provided stored serum samples for serum CrAg testing. The BED study was conducted from April – December 2009 in Hanoi, Ho Chi Minh City (HCMC), Haiphong, and Quang Ninh; eligible enrollees were ART-naïve adults (≥18 years of age) with documented HIV infection for at least one year [22] presenting to outpatient clinics (OPCs) for HIV care. Patients from the BED study were excluded from this sub-study if they had a recent or current diagnosis of CM. The HIV-DR study began in November 2009, and is scheduled for completion in March 2013. Enrollees were adults (≥18 years of age) with HIV infection initiating a first-line ART regimen at eight OPCs in northern and southern Vietnam. Sera collected at study enrollment from patients with CD4<100 cells/mm^3^ in both studies were stored at −20°C to −80°C at the National Institute for Hygiene and Epidemiology (NIHE) in Hanoi and Pasteur Institute (PI) in HCMC, and were retrieved for CrAg LFA testing in this sub-study during 2012. Training of laboratory staff to perform and interpret the LFA was completed before CrAg testing at both sites. Institutional review board (IRB) approval for this study was obtained from the IRB at the Centers for Disease Control and Prevention and the Vietnam Ministry of Health. Surviving patients from both the BED-assay and HIV-DR studies gave written informed consent after sera collection to have their remnant sera tested for CrAg in this sub-study. Deceased patients were exempt from the informed consent process and their sera were also tested for CrAg.

### Specimen Data Analysis

All analysis was done in SAS 9.3 (SAS Institute Inc., Cary, NC, USA). The χ^2^ or Fisher’s exact tests (when cell sizes<5) were used to compare proportions. The Wilcoxon rank-sum test was performed for comparison of medians.

### Definitions

For the cost-effectiveness model, CM is defined as a positive lumbar puncture (LP) in any patient, regardless of symptoms. LP positivity includes evidence of cryptococcosis in the cerebrospinal fluid (CSF) by any method (antigen test, India Ink, or culture). A positive symptom screen for CM is defined as one or more of the following symptoms: fever, headache, blurry vision, confusion, neck stiffness/soreness, or sensitivity to light. Isolated serum CrAg positivity, or asymptomatic cryptococcal antigenemia, is defined as a positive serum LFA result for CrAg in the absence of a positive symptom screen or positive LP. LP refusal includes all patients who do not have an LP performed after referral. We used WHO’s Choosing Interventions that are Cost Effective (CHOICE) guidelines [23], [24] to evaluate cost-effectiveness.

### Cost-effectiveness Evaluation and Assumptions

We evaluated the incremental cost-effectiveness of a CrAg screening program among Vietnamese HIV-infected patients newly presenting for ART at an OPC, and compared it to the current standard of care for HIV-infected patients in Vietnam (no cryptococcal screening, only treatment for symptomatic CM) [25]. General assumptions of this model include the following: 1) no patient treated for isolated positive serum CrAg develops CM; 2) loss to follow-up is negligible; and 3) the sensitivity and specificity of the LFA are 100%. Outcomes evaluated included number needed to screen to prevent one case of CM; number needed to screen to prevent one CM death; and (undiscounted) cost per life-year gained.

### Model Flow

**Figure 1:**
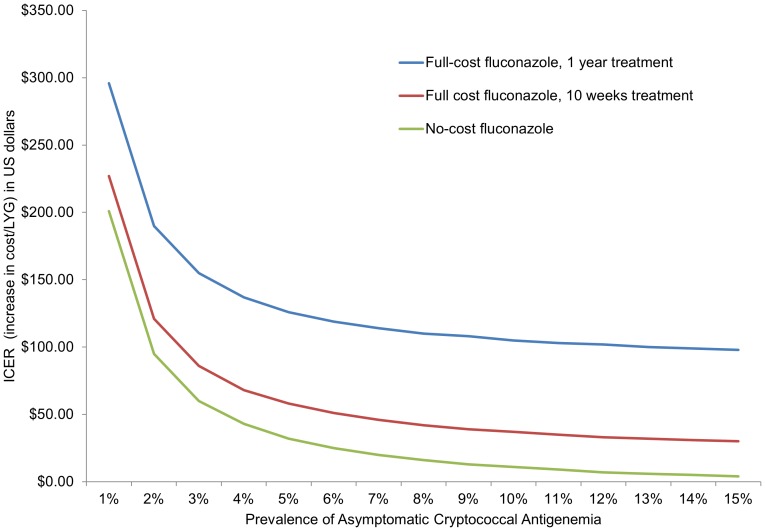
Cryptococcal screening algorithm. This algorithm, adapted from the South African national cryptococcal screening program, shows the flow of evaluation of HIV-infected patients in a model cryptococcal screening program. First, all patients with CD4<100 cells/mm^3^ undergo cryptococcal antigen (CrAg) testing. Those patients with a positive CrAg test then return to clinic for a symptom screening, followed by lumbar puncture referral for patients with a positive symptom screen. The algorithm also outlines the appropriate treatment for different patients within the screening program. Patients who have a negative CrAg test do not receive fluconazole but instead initiate antiretroviral therapy (ART) as per usual clinic practice. Patients who have a positive CrAg test and have a negative symptom screen or who have a positive symptom screen but negative lumbar puncture are treated with oral fluconazole for prevention of cryptococcal meningitis. Patients who have a positive symptoms screen and positive lumbar puncture are treated for cryptococcal meningitis according to Vietnam’s national HIV guidelines, with amphotericin B followed by fluconazole. Persons who have a positive symptom screen but refuse lumbar puncture are treated presumptively for cryptococcal meningitis. All CrAg-positive patients are initiated on ART after a minimum of two weeks of cryptococcal treatment (either for asymptomatic antigenemia, diagnosed, or presumptive cryptococcal meningitis).

Figure 1 demonstrates the flow of patients through a model cryptococcal screening program, adapted from the national cryptococcal screening program in South Africa [26]. Based on the model, ART-naïve patients presenting to an OPC with CD4<100 cells/mm^3^ receive serum CrAg testing; if negative, patients are initiated on ART as per Vietnam national HIV guidelines [25] and receive no further CrAg testing or treatment. Serum CrAg-positive patients return for an additional clinic visit and symptom screen. Patients with a negative symptom screen will receive treatment for isolated serum CrAg positivity (oral fluconazole, per WHO recommendations [13]). Patients with a positive symptom screen are referred for LP at a designated hospital. If the LP is positive, the patient will be treated for CM according to current Vietnamese national HIV guidelines [25]. If the LP is negative, the patient will be treated for isolated serum CrAg positivity with oral fluconazole [13]. If a symptomatic patient refuses LP, he/she will be referred for empiric CM treatment (Figure 1).

### Costs

All costs and assumptions are included in Tables 1 and 2. The LFA cost was derived from the manufacturer’s recommended retail price for resource-limited settings [27]. Per-unit costs for fluconazole and amphotericin B were provided by the HCMC People’s Action Committee (PAC), the administrative body that oversees delivery of HIV/AIDS care in HCMC. CM treatment was based on current Vietnamese national HIV guidelines [25], and includes a 14-day course of amphotericin B followed by fluconazole until immune reconstitution. Treatment of isolated serum CrAg positivity was based on the WHO guidelines for diagnosis, prevention and management of cryptococcal disease, and includes one year of fluconazole: 800 mg/day for two weeks, followed by 400 mg/day for eight weeks, followed by 200 mg/day maintenance [13]. Clinical cost estimates, including the cost of clinic visits, personnel time, diagnostic tests (e.g. LP, culture), hospitalization, and follow-up were provided by the National Hospital for Tropical Diseases (NHTD) in Hanoi (Dr. Cao Thuy, personal communication). These costs were compared with those at the Tropical Disease Hospital in HCMC and found to be equivalent. All direct costs, including physician care, hospitalization, and nursing care, were incorporated into the model. Indirect costs were not included in this analysis. Costs of CD4 testing, the initial clinic visit for enrollment in HIV care, and subsequent cost of ART are not included, as those do not represent additional costs to the system. Costs were not discounted in this model as they accrued over less than a one-year time horizon.

**Table 1:** Costs associated with cryptococcal meningitis diagnosis, treatment and a cryptococcal screening program in Vietnam, in US dollars.

Item	Unit Cost	# Units/Days	Total Cost
**Costs for all screened patients**			
LFA test	$4.13	1	$4.13
**Additional costs for all serum CrAg-positive patients**			
Return clinic visit for symptom screen	$5.00	1	$5.00
**Additional costs for symptom screen positive patients**			
Lumbar puncture	$1.68	1	$1.68
Testing on CSF*	$21.62	1	$21.62
**Additional costs for LP positive/Cost to treat Cryptococcal Meningitis**			
Lumbar puncture	$1.68	5	$8.40
Testing on CSF*	$21.62	2	$43.24
Hospitalization in ICU	$16.80	7	$117.60
Hospitalization in ward bed	$2.40	13	$31.20
Laboratory costs	$4.81	10	$48.10
Care services in ICU	$8.75	7	$61.25
Amphotericin IV	$8.17	14	$114.38
Fluconazole 800 mg/day	$2.31	56	$129.36
Fluconazole 200 mg/day	$0.58	295	$170.10
**Additional costs for serum CrAg-positive, LP-negative patients**			
Fluconazole 800 mg/day	$2.31	14	$32.34
Fluconazole 400 mg/day	$1.17	56	$64.52
Fluconazole 200 mg/day	$0.58	295	$170.10

* Inclusive of: CSF culture, cell count, glucose, protein, India Ink, and LFA.

**Table 2:** Assumptions of a cost-effectiveness model for cryptococcal screening in Vietnam.

Assumption	Value	Source
**General**		
Average age of CM diagnosis	28	[29]
Years of life gained if a person does not die of CM	25	[30], [31]
% of antigenemic patients who get CM if no antifungal treatment	30%	[11]
**Screening**		
% of patients with a positive symptom screen	50%	Personal communication*
% of patients who refuse an LP	5%	Personal communication*
% of positive LPs among serum CrAg-positive patients	50%	[9], [28]
Six-month mortality among isolated serum CrAg-positive patients	15%	[12], [17]
Six-month mortality among CM patients	30%	[5], [6] **
Six-month mortality among serum CrAg-negative patients	5%	[10]
Six-month mortality among LP refusers	20%	Extrapolated
**No Screening**		
Six-month mortality among CM patients	45%	[5], [6]
Six-month mortality among non-CM patients	10%	[10] ***

* Dr. Cao Thuy, physician.

** Assumed to be slightly lower than mortality among CM patients under existing standard of care, due to earlier diagnosis and treatment.

*** Assumed to be slightly higher than mortality among serum CrAg-negative patients under a screening scenario.

### Assumptions under Non-screening Scenario

Table 2 outlines assumptions used in the cost-effectiveness model. Six-month mortality estimates among CM patients in a non-screening situation (i.e., current standard of care) were based on reports from existing literature [5], [6]. Six-month mortality among non-CM patients in a non-screening scenario was assumed to be 10%, slightly higher than mortality among serum CrAg-negative patients reported in a previous study (under a screening scenario) [10]. The proportion of unscreened patients who progress to meningitis was derived from existing literature [11].

### Assumptions under Screening Scenario

With screening, six-month CM mortality among patients diagnosed with cryptococcal meningitis was assumed to be slightly lower (due to presumed diagnosis and treatment of disease at an earlier stage) than CM mortality under the non-screening scenario (Table 2). Currently, no data are available regarding mortality among isolated serum CrAg-positive patients treated with a standardized, WHO-recommended fluconazole regimen, so we assumed their mortality to be slightly lower than reported rates of mortality in serum CrAg-positive patients, all of whom were treated with a non-standardized regimen [12], [17]. Six-month mortality among serum CrAg-negative patients was derived from a previous study [10]. Six-month mortality among serum CrAg-positive patients who refuse LP was assumed to be a value intermediate between the estimated mortality in isolated serum CrAg-positive and CM patients.

The estimated proportion of serum CrAg-positive patients with a positive LP after a symptom screen was based on reports in the literature [9], [28]. The proportion of patients with a positive symptom screen and the frequency of LP refusals were estimated through consultation with in-country physicians with experience in HIV care, including care for persons with CM (Dr. Cao Thuy, personal communication) Average age at diagnosis of CM was derived from the literature [29], as was life expectancy of HIV-infected persons on ART with CD4<100 cells/mm^3^ [30], [31].

### Data Analysis: Cost-effectiveness Model

The number of patients needed to screen (NNS) to prevent one case of CM or one death from CM was calculated as:
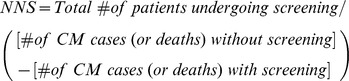



Undiscounted life-years gained (LYG) were calculated by multiplying excess deaths from CM by years of life gained when a screened patient did not die from CM. We calculated the incremental cost effectiveness ratio (ICER) of screening as the excess cost associated with screening divided by the number of LYG through screening.

### Alternate Models: Cost and Length of Fluconazole Treatment

Currently, WHO-recommended length of fluconazole maintenance treatment for isolated serum CrAg-positive patients (until immune reconstitution) is based on expert opinion. In our model, we assumed the total time required for treatment of an isolated serum CrAg-positive patient was one year of fluconazole therapy. However, at least one observational study has demonstrated a survival benefit with as few as 2–4 weeks of fluconazole therapy [12]; other clinicians have suggested that fluconazole treatment past 10 weeks may be unnecessary [15]. Due to this uncertainty, fluconazole treatment lengths of 10 weeks and 1 year were both evaluated in this analysis under the same presumed effectiveness in preventing CM. In addition, because some countries, including Vietnam, receive no-cost fluconazole for CM treatment through Pfizer’s Diflucan Partnership Program (DPP) [32], we calculated cost per LYG for full-cost (at 10 weeks and 1 year of treatment) and no-cost fluconazole. All cost-effectiveness modeling was done in MS Excel 2010 (Microsoft Corp., Redmond, WA, USA).

### Sensitivity Analysis

To evaluate the sensitivity of the model, we calculated cost per LYG while varying CrAg prevalences (2%, 4%, and 6%), LFA test costs (+/−50%), fluconazole costs (+/−50%), percentage of serum CrAg-positive patients with a positive symptom screen (+/−20%), percentage of patients with a positive LP (+/−20%), six-month mortality rate among screened patients with CM (+/−50%), and with discounting of health benefits at 3% and 5%.

## Results

### Serum CrAg Prevalence Overall, and by Region

Two hundred twenty-six patients with CD4<100 cells/mm^3^ were evaluated for serum CrAg: 142 from the BED study and 84 from the HIV-DR study. Median CD4 count was 40 (range: 0–99) cells/mm^3^, and was significantly lower in the South compared with the North (28 vs. 51 cells/mm^3^, p<0.0001). Nine (4%; 95% CI 2–7%) serum CrAg-positive samples were identified; two (2%, 95% CI 0–6%) of 104 specimens from patients in North Vietnam and seven (6%, 95% CI 3–11%) of 122 specimens from patients in South Vietnam (p = .18). Median CD4 count was not different between CrAg-positive and CrAg-negative persons (39 vs. 40 cells/mm^3^, p = 0.86). Five (56%) of the nine CrAg-positive tests occurred in persons with a CD4<50 cells/mm^3^ (Table 3). No patient enrolled in the BED study had a recent or current diagnosis of CM at the time of enrollment; these data were not available for patients from the HIV-DR study.

**Table 3:** CrAg-positive prevalence by CD4 count and region of Vietnam.

CD4 count(cells/mm^3^)	North	South	Total
**50–99**	1/53 (2%)	3/33 (9%)	4/86 (5%)
**<50**	1/51 (2%)	4/89 (4%)	5/140 (4%)
**Total**	2/104 (2%)	7/122 (6%)	9/226 (4%)

### Cost-effectiveness of Screening

**Figure 2:**
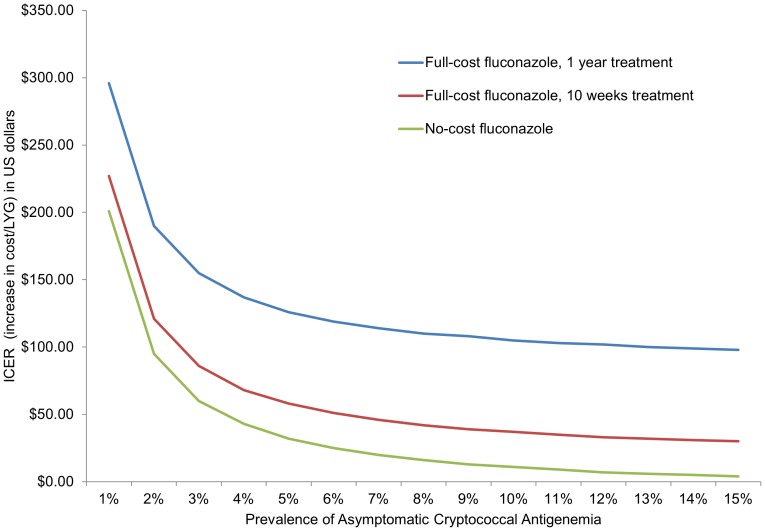
Cost-effectiveness of cryptococcal screening at a range of prevalences and cost scenarios. This graph has prevalence of asymptomatic cryptococcal antigenemia on the x-axis and incremental cost-effectiveness ratio (ICER) (increased cost per life-year gained [LYG] in US dollars) on the y-axis. The blue line represents the cost curve of full-cost fluconazole for one year of treatment. The red line represents the cost curve of full-cost fluconazole for ten weeks of treatment; the green line represents the cost curve of if fluconazole is obtained at no-cost. For Vietnam, the World Health Organization considers any intervention with an ICER under $6,948 to be ‘highly cost-effective’. This graph shows that cryptococcal screening, at any prevalence and under each of the three cost scenarios, should be considered a highly cost-effective intervention in Vietnam.

With a full year of fluconazole treatment for isolated serum CrAg-positive patients, the ICER for CrAg screening at a serum CrAg prevalence of 4% is estimated at $137/LYG; at a prevalence of 2% it is $190/LYG and at a prevalence of 6% is it $119/LYG. For a limited 10-week course of fluconazole, the estimated ICER of screening was $68/LYG at a serum CrAg-positive prevalence of 4%, $121/LYG at 2%, and $51/LYG at 6%. When fluconazole costs are removed from the model (i.e., no-cost fluconazole), the estimated ICER of screening is $43/LYG at a serum CrAg-positive prevalence of 4%, $95/LYG at 2%, and $25/LYG at 6%. Figure 2 shows the ICER at varying serum CrAg prevalences and under different cost and fluconazole treatment length scenarios. WHO considers an intervention to be ‘very cost-effective’ if the ICER is less than the gross domestic product (GDP) per capita of the WHO region (for Vietnam, $6,948) [23], [24].

At a serum CrAg-positive prevalence of 4%, 228 patients with CD4<100 cells/mm^3^ need to be screened to prevent one case of CM, and 321 need to be screened to prevent one death from CM. These values vary with the prevalence of cryptococcal disease (Table 4).

**Table 4:** Number Needed to Screen (NNS), by prevalence of serum CrAg-positive persons in Vietnam.

Prevalence	NNS to prevent one case of CM	NNS to prevent one death from CM
2%	455	641
4%	228	321
6%	151	214

### Sensitivity Analysis

The cost-effectiveness of screening for CrAg is inversely related to LFA costs, fluconazole costs, the six-month CM mortality rate among screened patients, the proportion of serum CrAg-positive patients with a positive symptom screen, the proportion of patients with a positive LP, and the discount rate of health benefits (Table 5). The cost-effectiveness of screening was sensitive to changes in six-month CM mortality among screened patients and the discount rate of health benefits, but relatively insensitive to changes in price of fluconazole, LFA cost, proportion of serum CrAg-positive patients with a positive symptom screen and proportion of patients with a positive LP (Table 5).

**Table 5:** Sensitivity analysis: variation in cost/LYG through screening by varied testing and drug costs, positive symptoms screen, LP rate, CM mortality rate and discount rate.

		LFA costs		Fluconazole costs		% of serum CrAg+ patients with a positive symptom screen		% (+) LP		6-mo. mortality of CM (screened)		Discounted health benefits	
CrAg (+) Prevalence	Base model	Reduced by 50%	Increased by 50%	Reduced by 50%	Increased by 50%	Reduced by 20%	Increased by 20%	Reduced by 20%	Increased by 20%	Reduced by 50%	Increased by 50%	3%	5%
**2%**	$190	$137	$243	$142	$237	$156	$235	$158	$231	$139	$299	$395	$649
**4%**	$137	$110	$163	$89	$184	$110	$173	$112	$170	$100	$215	$285	$468
**6%**	$119	$101	$137	$72	$166	$94	$152	$96	$150	$87	$187	$248	$407

## Discussion

Treatment of patients with HIV-associated CM is extremely costly and is associated with poor outcomes in resource-limited settings, such as Vietnam. Even under optimal circumstances, mortality can be as high as 15% [7]. To reduce CM deaths, the WHO has recommended consideration of screening HIV-infected, ART-naïve patients with CD4<100 cells/mm^3^ for cryptococcal disease in areas with a high prevalence of *Cryptococcus* infection [13]. In this analysis, we found serum CrAg to be present in 2–6% of HIV-infected patients with CD4<100 cells/mm^3^ in Vietnam. At these prevalences, a cryptococcal screening program in Vietnam would cost less than $190 per life-year gained.

The lower prevalence of serum CrAg positivity we observed in Vietnam, compared to neighboring countries, [9], [17] might be due to differences in the study populations. In Cambodia, serum CrAg prevalence among persons with CD4<100 cells/mm^3^ was 21% [17]; however, the population tested was 50% inpatients and most already had symptomatic CM [17]. CrAg screening, as suggested by the WHO, is intended to target HIV-infected persons with CD4<100 cells/mm^3^ who are healthy enough to present for initiation of ART. Our prevalence estimates are closer to those found among HIV-infected patients presenting for ART initiation at ambulatory care clinics in sub-Saharan Africa (6% in Uganda and 7% in Kenya) [10], [33]. However, one study in Thailand which evaluated asymptomatic, ART-naïve patients found a CrAg-prevalence of 13% [9], suggesting that there may be real differences in prevalence of cryptococcal disease between regions in Southeast Asia, perhaps due to environmental or host factors. It is worth noting that all currently available estimates of CrAg prevalence in Southeast Asia are based on small samples of patients; data from larger studies would provide more robust estimates with which to compare regional differences. The lower prevalence of serum CrAg positivity in northern versus southern Vietnam, while not statistically significant and based on small patient numbers, is consistent with published findings from studies of OIs in major referral hospitals in Hanoi [34] and HCMC [2], [29] which documented less CM in the north than the south of Vietnam.

Interest in primary cryptococcal prophylaxis with an oral azole for all high-risk patients, though initially supported by the WHO [35], has declined in recent years due to the expense, concerns about resistance, and the failure to consistently document a survival benefit [36], [37], [38], [39], [40]; targeted screening was considered likely to be a more cost-effective approach. Several articles have been published on cryptococcal screening in recent years [10], [11], [12], [15], [16], [17], [26], [33], [41], [42], resulting in increased country-level interest in implementation of CrAg screening programs. South Africa, with the largest population of HIV-infected persons in the world, began a nationwide rollout of cryptococcal screening at outpatient clinics in mid-2012, integrating screening into their national health plan [26]. Given the increasing interest in CrAg screening in resource-limited settings, a detailed cost-effectiveness evaluation, such as the one presented here, is essential for program planning, appropriate budgeting and allocation of limited resources, and subsequent program monitoring and evaluation.

We chose cost/LYG as our metric of evaluation in this paper for several reasons. First, although data on CM mortality in resource-limited settings are already fairly well-established [1], disability due to CM is poorly quantitated in these same areas, making metrics such as DALYs or QALYs potentially less valid and less meaningful than LYG, which includes only effects on mortality. Additionally, cost/LYG is a relatively easy and transparent method for measuring population health for a disease with high mortality, such as CM, and is easily comparable across interventions. Using cost/LYG, we found that the incremental cost-effectiveness of cryptococcal screening in Vietnam varied widely, from $4–296/LYG, based on serum CrAg prevalence and inclusion of fluconazole costs (Figure 2). WHO-CHOICE guidelines consider a very cost-effective intervention to be one in which the incremental cost-effectiveness ratio is less than the GDP per capita in the respective WHO region [23]. Vietnam is in the West Pacific Region (WPRO) B region which has an estimated GDP per capita of $6,948 [24], making the implementation of a cryptococcal screening program very cost-effective, by WHO standards, at all CrAg prevalence estimates evaluated in this study. In 2011, the World Bank estimate of per capita GDP in Vietnam was $1,411 [43]; while significantly lower than the WPRO B per capita GDP, it is still within a range in which a cryptococcal screening program would be considered very cost-effective by WHO. In addition, cryptococcal screening compares favorably to other HIV care interventions: cost-effectiveness studies of cotrimoxazole prophylaxis show a cost/LYG of $150–$1180 [44], while studies of ART report a cost/LYG of $430 to >$1,000 [45], [46], [47].

It is worth noting that previous evaluations of cost-effectiveness of CrAg screening either have not included fluconazole costs [12] or have included a much-reduced regimen, in both dosage and length of treatment, compared with the WHO-recommended regimen [16]. Fluconazole maintenance therapy for isolated serum CrAg-positive patients, as recommended in the WHO guidelines, comprises a substantial proportion of the cost of a screening program, due to the recommendation to treat until immune reconstitution (assumed to be 1 year of treatment in this analysis). While inclusion of the WHO-recommended fluconazole regimen does not make cryptococcal screening cost-ineffective in our analysis, it does increase costs substantially, which warrants consideration in the implementation planning of this and other large-scale cryptococcal screening programs. Our values for the NNS to avoid one case and one death from CM are slightly higher than other estimates in the literature [11], [12], likely due to differences in the CrAg prevalence between Vietnam and sub-Saharan Africa, where other studies reporting NNS were conducted.

This study includes the following limitations. First, all costs and most assumptions were based on data from Vietnam or Southeast Asia, and may not be generalizable to other regions of the world. Our estimates of CrAg prevalence are based on a limited number of patients accessing care at clinics in two regions of Vietnam, and may not be generalizable to the entire country; larger studies of HIV-infected persons in multiple regions of Vietnam should be undertaken to better understand the country-wide burden of cryptococcal disease. Second, the flow and management of patients through our screening model represent ideal practice; real-world practices may be different. Third, we lacked clinical data for participants from the HIV-DR study and were thus unable to rule out current or recent CM in those patients. Finally, most of the assumptions underpinning this model were derived from small observational studies, including data on the mortality benefit from early treatment of CrAg-positive persons with fluconazole. Further research, including randomized controlled trials of fluconazole for early cryptococcal disease, is needed to confirm these preliminary data.

To the best of our knowledge, this is the first study to estimate the prevalence of serum CrAg positivity in Vietnam. It is also the first evaluation of the cost-effectiveness of cryptococcal screening according to treatment outlined in the WHO guidelines for diagnosis, prevention and management of cryptococcal disease. Based on our estimates of CrAg prevalence in Vietnam, implementation of a cryptococcal screening program is both indicated and highly cost-effective in Vietnam.
